# Cases of Adverse Reaction to Psychotropic Drugs and Possible Association with Pharmacogenetics 

**DOI:** 10.3390/jpm2040149

**Published:** 2012-10-01

**Authors:** Irina Piatkov, Trudi Jones, Mark McLean

**Affiliations:** University of Western Sydney Clinic and Research Centre Blacktown, Western Sydney Local Health District, Blacktown 2148, NSW, Australia; E-Mails: trudi.jones@swahs.health.nsw.gov.au (T.J.); m.mclean@uws.edu.au (M.M.)

**Keywords:** venlafaxine, nortriptyline, suicide, adverse effects, genetic polymorphisms, cytochrome P450, pharmacogenetics

## Abstract

Thousands of samples for pharmacogenetic tests have been analysed in our laboratory since its establishment. In this article we describe some of the most interesting cases of CYP poor metabolisers associated with adverse reactions to psychotropic drugs. Prevention of disease/illness, including Adverse Drug Reaction (ADR), is an aim of modern medicine. Scientific data supports the fact that evaluation of drug toxicology includes several factors, one of which is genetic variations in pharmacodynamics and pharmacokinetics of drug pathways. These variations are only a part of toxicity evaluation, however, even if it would help to prevent only a small percentage of patients from suffering adverse drug reactions, especially life threatening ADRs, pharmacogenetic testing should play a significant role in any modern psychopharmacologic practice. Medical practitioners should also consider the use of other medications or alternative dosing strategies for drugs in patients identified as altered metabolisers. This will promise not only better and safer treatments for patients, but also potentially lowering overall healthcare costs.

## 1. Introduction

The variation in individual responses to psychotropic drug treatment remains a critical problem in the management of psychotic disorders. The prescription of antidepressants has increased rapidly in recent years. Epidemiological studies suggest that depression is the second most significant cause of disability in the health care arena [1]. Up to 10% of the population at any one point in time, may be depressed and up to 45% of the population may, at some point during their lifetime, suffer from a depressive episode. Although most patients will experience remission, some develop drug-induced adverse effects that can range from troublesome to life threatening [2]. 

Our Laboratory was established in 2005 and since that time we have analyzed thousands of samples for cytochrome P450 and other genes involved in drug metabolism. From our experience with pharmacogenetics, we have observed several adverse drug reactions in patients with poor metabolizer genotype and some of the most severe cases are described here after consent to publish their de-identified genotyping results were obtained.

*Case 1.* Two patients experienced single events of violent psychosis after the tricyclic antidepressant nortriptyline was prescribed. Both patients were female, one Caucasian and the other Asian. Both were drug naive with no history of any prescription or illicit drug use prior to the initiation of treatment. Both patients had neither history of antisocial behaviour nor any family history of psychosis. No recurrence of the psychotic episode was observed after drug withdrawal. 

Patient A: Female, 40 years old, Caucasian, with no history of violent behaviour or psychotic disorders in her family. She suffered from stress-induced mood changes. Her doctor recognized depression and prescribed nortriptyline, 10 mg 4 times/day. According to her husband, the next day she started to behave abnormally, complaining that she was hearing strange voices. On the third day, she experienced a severe psychotic episode which resulted in the loss of her child. As the patient herself described, she felt dizzy, disorientated and was suffering from delusions, and had no memory of what had happened. Since withdrawal of the drug, she has not experienced another episode. 

Patient B: Female, 17 years old, Asian, with no history of violent behavior or psychotic disorders in her family. She suffered from stress during her high school exams. Depression was recognized by her doctor and nortriptyline was prescribed, 10 mg 3 times/day. After two weeks, she was admitted to the hospital with a severe psychotic episode. Since withdrawal of the drug, she has not experienced another episode. 

*Case 2.* Seven members of Family X were analyzed. Both parents are Caucasians. Three family members had been diagnosed with depression and venlafaxine was prescribed. However, none of the family members can recall their prescription dosages, with the exception of the forensic case, where venlafaxine was detected in blood (4.5 mg/L). All three then experienced severe adverse drug reactions, which led to the tragic loss of one of the family. As per their own description, they experienced hallucinations, dysphoria, restlessness, suicidal ideation and suicide attempts. No herbal or supplementary medications were implemented, according to patient’s reports, but coincidentally an allergic reaction to eggs is common in the family. 

Adverse Drug Reaction (ADR), which manifests as homicide, suicide or psychotic episodes, is the subject of debate in both the medical and scientific community.

Tricyclic antidepressants (TCAs) have a moderate therapeutic index, as they produce significant adverse effects at therapeutic concentrations and are dangerous in overdose. Significant side effects of TCAs are common and their prevalence is estimated as high as 5%, while acute poisoning with TCAs is potentially life-threatening [3]. Central nervous system manifestations of toxicity include agitation, stupor, coma, seizure and manic excitement. The plasma level correlates poorly with the severity of symptoms and peak blood levels over 1,000 ng/mL have a higher risk of cardiac and CNS toxicity [4,5]. TCAs exist as tertiary or secondary amines and the tertiary forms are metabolized to secondary amines. Both tertiary and secondary amines are active, as are some of the subsequent hydroxylated metabolites. The tertiary amines are metabolized by many P450s, whereas the secondary amines are largely metabolized by CYP2D6. The major metabolite produced by CYP2D6, E-10-hydroxynortriptyline, has approximately half the potency of the parent drug in inhibiting noradrenaline reuptake and greatly reduced anticholinergic activity. It is often present at comparable (or higher) concentrations to the parent drug and may contribute to the antidepressant effects [6].

Differences were reported in the nortriptyline clearance between the groups of dissimilar CYP2D6 genotypes in diverse ethnic populations and it was demonstrated that dysfunctional CYP2D6 alleles had a greater risk of side effects [7,8,9,10,11].

Adverse drug reactions or treatment resistance to venlafaxine are described in some publications [12,13,14,15,16,17,18,19,20,21,22], including Serotonin Syndrome induced by low-dose venlafaxine [19] and [13]. Chan *et al.* [23] reported that those who ingested venlafaxine were more likely to become confused and have mydriasis, than those who took SSRIs. Compared with SSRI self-poisoners, patients who deliberately ingested venlafaxine were more likely to exhibit serious suicide intent. Venlafaxine is an antidepressant that is biotransformed to the active metabolite *O*-desmethylvenlafaxine, primarily by the CYP450 enzymes. Research in the pharmacogenetics of antidepressants, venlafaxine amongst them, demonstrated that polymorphic variations in the CYP450 genotype could be used in dosage adjustment in treatment resistant patients [24,25,26]. 

In addition to its role in the metabolism of drugs, CYP2D6 is highly expressed in several regions of the brain, such as the hippocampus, thalamus, hypothalamus and the cortex, and plays a role in the dopamine pathway [27,28,29]. However, cross-study reproducibility is difficult due to complicity of psychopathology and neurotoxicity manifestation.

## 2. Method

Previously we have described Cytochrome P450 genotyping results for 11 samples from deceased persons who committed suicide during treatment on venlafaxine [30]. Authorisation from the NSW State Coroner to perform post-mortem genetic testing was obtained for 10 samples from deceased persons who committed suicide during treatment on venlafaxine. The family of the one of the deceased persons in this study then gave their consent to publish their genotyping results, which were obtained previously. Ethics approval was obtained from Sydney South West Area Health Service Ethics Committee. 

Patient consent was obtained from one of the nortriptyline cases ten years ago, but none of the patients are currently available for confirmation of their consent. However, we think that these cases are a good illustration for the necessity of early prevention biomarkers and only deidentified information is used in the article. 

DNA was extracted from swabs collected by the participants. The variant alleles of CYP2D6*2 (2850C > T), *3 (2549delA), *4 (1846G > A), *5 deletion, *10 (100C > T), *17 (1023C > T, 2850C > T), *41(2988G > A); CYP2C9*2 (430C > T), *3 (1075A > C) and CYP2C19*2 (681G > A), *3 (636G > A), *17 (−806C→T) that affect the function of cytochrome enzymes were genotyped at our Research Laboratory. DNA was extracted from swabs using the manufacturer’s protocol for the QIAGEN EZ1 BioRobot system. The genotyping method involves specific restriction enzyme digestion of amplified PCR products or tetra-primer allele-specific amplification PCR. Fragment analysis is based on capillary electrophoresis, the methodology of which is described in detail in our previous publication [31].

## 3. Results and Discussion

### 3.1. Results

In patient A, pharmacogenetic tests revealed a loss-of-function CYP2D6 *4/*41 polymorphism and in patient B, tests revealed a CYP2D6*10/*10 polymorphism with diminished enzyme activity.

Results for Family X (case 2 in Introduction) revealed that all family members have at least one copy of the loss-of-function cytochrome P450 allele (Table 1).

The majority of Family X members have multiple loss-of-function polymorphisms, which include CYP2D6 and CYP2C9 genes. These genes encode several enzymes of the metabolic pathway of venlafaxine. A combined effect of diminished enzyme activities on the venlafaxine metabolic pathway could contribute to the impairment of venlafaxine metabolism.

**Table 1:** Family X (case 2) genotype results.

	Age	Loss of Function CYP Polymorphisms detected	Adverse Drug Reaction on Venlafaxine	Adverse drug reaction manifestation on Venlafaxine	Other known ADRs or allergies
Father	59	CYP2C9*2; CYP2D6*4/*10	no venlafaxine taken		egg allergy
Mother	56	CYP2C9*2	severe	hallucinations, dysphoria, restlessness, suicidal ideation	
Daughter 1	36	CYP2C9*2/*2; CYP2D6*4/*10	no venlafaxine taken		
Son	26	CYP2D6*4/*10	extremely severe	dysphoria, restlessness, suicidal ideation, suicide	
Daughter 2	26	CYP2C9*2; CYP2D6*4/*10	no venlafaxine taken		
Daughter 3	23	CYP2C9*2	no venlafaxine taken		egg allergy
Daughter 4	21	CYP2D6*4/*10	extremely severe	hallucinations, dysphoria, restlessness, suicidal ideation, suicide attempt	ADR on fluoxetine, egg allergy

### 3.2. Discussion

Psychotropic medications have been associated with a variety of adverse drug reactions, including neurotoxicity development. Variability in the reaction to medication may be due to age, gender, morbidity, co-medication, food components, smoking and environmental factors. However, polymorphisms present in genes, are responsible for most of the variations. Pharmacokinetics and pharmacodynamics pathways candidate gene approaches have succeeded in the identification of several genetic factors influencing treatment response. CYP enzymes, dopamine and serotonin gene variants have been linked with treatment-associated side effects of psychotropic drugs [32,33].

The most commonly studied cytochrome P450 enzymes include 2D6, 2C19 and 2C9. Polymorphisms and gene duplications in these enzymes account for the most frequent variations in Phase I metabolism of drugs, since nearly 80% of all drugs in use today, along with most psychotropics, are metabolized via these pathways [34]. According to our data published in “Pharmacogenetics” [31] and other publications [35,36], patients with drug-induced akathisia have a higher prevalence of abnormal metabolizer genotypes. 

Venlafaxine and nortriptyline are metabolized to their active metabolites by cytochrome P450 enzymes: 2D6 (CYP2D6), 2C9 (CYP2C9) and 2C19 (CYP2C19), which are highly genetically polymorphic. Allelic variants can cause reduced activity, loss of cytochrome enzyme function or ultra rapid enzyme activity in comparison with a wild type gene. Certain polymorphisms are associated with reduced or absent Cytochrome P450 enzyme activity and low metabolite levels in treated patients. Individual variations in activity of metabolic enzymes can affect drug tissue distribution and therapeutic/toxic concentration, which in turn can influence a patient’s response to treatment or toxicity development [37,38,39,40]. 

Cytochrome P450 (CYP450) is a superfamily of proteins that play an important role in the Phase I oxidative metabolism of many endogenous and exogenous compounds. Altered CYP450 function can lead to the build-up of toxic and harmful substances in the bloodstream, which in turn lead to severe side effects and in combination with drugs to adverse drug reactions. The association between CYP450 polymorphisms and psychotropic ADRs could also be explained by variations in drug metabolism, interactions with endogenous and exogenous compounds, or due to cytochrome P450’s participation in dopamine metabolism. Interactions between substances metabolized through the CYP450 system and an individual’s variation in enzyme activity should also be considered in toxicity interpretation along with other factors.

The authors consider that Phase 1 cytochrome P450 loss-of-function polymorphisms should be included in patient record warning labels, similar to allergies, as a warning that an individual patient is susceptible to adverse drug reactions or has a limited capacity for elimination of toxins, leading to the development of allergies or toxicity.

Described results demonstrate the importance of individual approaches in psychotropic drug prescriptions and recommendations for ADR prevention. According to all patients involved, none of them had been informed about venlafaxine or nortriptyline adverse effects prior to treatment. We could speculate that this simple action could have prevented the horrific episodes associated with these cases and the loss of two young lives. 

Psychotropic drug prescribers should consider treatment-resistant patients as potential abnormal metabolisers, as in the abnormal metaboliser population, somatic symptoms associated with psychiatric diagnoses may in fact be caused by medication intolerance.

One of the aims of rapidly developing personalised medicine is prevention. Educational programs like suicide prevention programs for medical practitioners, nurses and caregivers should include a pharmacogenetics curriculum. Patients and their caregivers should be informed about ADR symptoms prior to treatment and ought to actively participate in a treatment regime.

## 4. Conclusions

Our results suggest an association between poor metabolizer genotypes and adverse reactions to drugs in the described patient cases. 

Prevention of disease/illness, including Adverse Drug Reaction (ADR), is an aim of modern medicine. However, despite the accumulation of pharmacogenetic data, its medical application is still a subject of debate and not enough educational programmes have been developed to introduce pharmacogenetics in patient care. 

Scientific data supports the fact that evaluation of drug toxicology includes several factors, together with genetic variations in pharmacodynamics and pharmacokinetics of drug pathways. Medical practitioners should consider the use of other medications or alternative dosing strategies for drugs in patients identified as altered metabolisers. We would like to cite one psychiatrist: “I have been sending you a few requests lately. I have been very encouraged by the response of one boy XXXXX whom you found was a poor metaboliser of 2D6. Up until then he had been on Risperdal and Zoloft. On the strength of the results, I left him on the Risperdal and changed the antidepressant to Escitalopram with very good results. The parents and the school have been delighted. He is a boy with severe developmental delay and behavioural difficulties, so any improvement in his behaviour is greatly appreciated by his parents and teachers. Thanks for your ongoing help”.

Implementation of pharmacogenetics in patient care promises not only better and safer treatments for patients, but also potentially lowering overall healthcare costs. Patients and their caregivers should always be informed about the symptoms prior to treatment and ought to actively participate in a treatment regime. Violence and suicide prevention programs for medical practitioners, nurses and caregivers should also include a pharmacogenetics curriculum.

## Supplementary Files

Supplementary File 1 PDF-Document (PDF, 182 KB)
