# Supplementary material for: Cases of Adverse Reaction to Psychotropic Drugs and Possible Association with Pharmacogenetics
Source: J Pers Med. 2012 Oct 1;2(4):149–57. doi: 10.3390/jpm2040149 (PMC4251377; doi:10.3390/jpm2040149)
Supplement: Supplementary File 1 — PDF-Document (PDF, 182 KB) [file jpm-02-00149-s001.pdf]

	Age	Loss of Function CYP Polymorphisms detected	Adverse Drug Reaction on Venlafaxine	Adverse drug reaction manifestation on Venlafaxine	Other known ADRs or allergies
Father	59	CYP2C9*2; CYP2D6*4/*10	no venlafaxine taken		egg allergy
Mother	56	CYP2C9*2	severe	hallucinations, dysphoria, restlessness, suicidal ideation	
Daughter 1	36	CYP2C9*2/*2; CYP2D6*4/*10	no venlafaxine taken		
Son	26	CYP2D6*4/*10	extremely severe	dysphoria, restlessness, suicidal ideation, suicide	
Daughter 2	26	CYP2C9*2; CYP2D6*4/*10	no venlafaxine taken		
Daughter 3	23	CYP2C9*2	no venlafaxine taken		egg allergy
Daughter 4	21	CYP2D6*4/*10	extremely severe	hallucinations, dysphoria, restlessness, suicidal ideation, suicide attempt	ADR on fluoxetine, egg allergy
